# Sensitivity Enhancement of Acetone Gas Sensor using Polyethylene Glycol/Multi-Walled Carbon Nanotubes Composite Sensing Film with Thermal Treatment

**DOI:** 10.3390/polym11030423

**Published:** 2019-03-05

**Authors:** Jin-Chern Chiou, Chin-Cheng Wu, Tse-Mei Lin

**Affiliations:** 1 Department of Electrical Engineering, National Chiao Tung University, 1001 University Road, Hsinchu City 30010, Taiwan; chiou@mail.nctu.edu.tw (J.-C.C.); s0450737.eed04g@g2.nctu.edu.tw (T.-M.L.); 2 Institute of Electrical and Control Engineering, National Chiao Tung University, 1001 University Road, Hsinchu City 30010, Taiwan

**Keywords:** acetone, polyethylene glycol, Multi-walled Carbon Nanotubes, thermostat, thermal distribution, temperature effect

## Abstract

There is a need to develop a chemiresistive gas sensor equipped with a thermostat over a wide area for the sensor, which can protect the sensor from the influence of ambient temperature due to the uniform temperature of the thermostat. In this paper, we demonstrated an acetone gas sensor based on a polyethylene glycol (PEG)/Multi-walled Carbon Nanotubes (MWCNTs) composite film, which was equipped with a thermostat. The sensor was operated at modest working temperatures for sensor sensitivity enhancement. The optimum design of the polyimide-based thermostat with widely uniform thermal distribution was investigated in detail. It was found that the temperature uniformity of the thermostat was achieved using double spiral geometry. The experimental results of the sensor response showed that the PEG/MWCNTs composite film with a moderate working temperature revealed a higher sensitivity than that without thermal treatment. Moreover, the sensing mechanisms of the PEG/MWCNTs composite gas sensor to acetone vapor were studied as well.

## 1. Introduction

Acetone [(CH_3_)_2_CO] as a disease-specific biomarker [1,2,3] and a flammable industrial solvent can be hazardous and life threatening when human beings are exposed to its high concentration. The permissible exposure limit-time weighted average (PEL-TWA) of acetone is 750 ppm (1800 mg/m^3^) and the permissible exposure limit-short time exposure limit (PEL-STEL) is 1000 ppm (2400 mg/m^3^) [4]. Therefore, the detection of acetone in the environment or in exhaled breath has attracted attention in order to develop relative wearable and portable gas sensors for fire accident prevention and diagnosis of diabetes.

Recent studies have made great efforts to develop miniaturized gas sensors for detecting acetone. Inorganic metal oxide-based sensors, such as iron oxide (Fe_2_O_3_/Fe_3_O_4_), zinc oxide (ZnO), tin oxide (SnO_2_), tungsten oxide (WO_3_), titanium oxide (TiO_2_), silicon oxide (SiO_2_), etc., operated at an elevated temperature to change oxygen stoichiometry and surface charge [5,6]. Zhang et al. proposed that the liquid phase separation method can be used to prepare ZnO/graphene (ZnO-G) hybrid composites. The fabricated ZnO/Graphene Composites film can detect 10–100 ppm acetone at 280 °C [7]. Patil et al. reported the preparation of two types of spinel MgFe_2_O_4_ thick films by sol–gel process. These films showed the fast response to acetone vapor operated at 350 °C and 450 °C [8]. Conductive polymers, including polyaniline (PANI), polypyrrole (PPy), polythiophene (PTh), poly(3,4-ethlenedioxiythiophene) (PEDOT) that utilize absorption/desorption by swelling and redox to be operated at room temperature [9,10] Do et al. reported using PANI with polymethylmethacrylate (PMMA) for sensing material, which revealed that a good response and sensitivity to low concentration acetone vapor [11]. Functional carbon nanotube (CNT) materials can interact with acetone and then induce charge transfer [12,13]. Leghrib et al. have successfully employed hybrid nanomaterials consisting of metal nanoclusters decorating MWCNTs with plasma treatment to detect benzene at ppb concentration [14]. Tan et al. studied the sensibility of the iron oxide (Fe_2_O_3_) nanopowder mixed with MWCNTs for acetone vapors [15]. There are some disadvantages that restrict their applications, including high power consumption of metal oxide, being non-conductive to desorption and low selectivity to different target gas of CNTs, and poor sensitivity of polymers.

Due to relatively easy preparation techniques and the combined advantages of both polymers and CNTs, polymer/CNT composite sensing films have been widely investigated regarding their selectivity, sensitivity, and low power consumption for volatile organic compounds (VOCs) detection [16]. Polymer/MWCNTs composites sensing film have attracted much attention due to their fast response and high sensitivity towards VOCs at room temperature. However, the characteristics of gas molecules, ambient temperature, and moisture seriously affect the sensitivity of the sensing performance, which are serious issues for researchers.

Several methods have been proposed to improve the response and recovery of the polymers, including the functionalized amino acid generation of CNTs in a polymer matrix [17], ultraviolet light (UV) illumination to facilitate the desorption of gas molecules [18], iron oxide and oxygen plasma functionalized multi-walled CNTs (MWCNTs) [19], and thermal treatment to enhance the fast recovery [20]. For a sensor equipped with a thermostat for gas sensing, thermal distribution affects the performance of the arranged polymer sensing element. The optimized design for thermal distribution is essential to the thermostat, which ensures each sensor element remains at the same operating temperature [21,22]. Therefore, the main objective of this paper is to study in detail the response characteristics of a PEG/MWCNTs composite at different operating temperatures. This method was utilized to enhance the sensitivity and the response of an acetone gas sensor.

In this work, we presented the optimum design of the thermostat in detail. We investigated the thermal distribution and power consumption of four different type geometries of heater coils regarding the thermostat. The optimum heater coil geometry according to simulation results was a double spiral type made of stainless steel. In addition, the response of the composite film sensors with a different quantity of PEG was studied.

## 2. Materials and Device Design

### 2.1. Materials

The deionized (DI) water-dispersed MWCNTs (XNM-HP-12050, concentration: 60 ppm) was purchased from XinNano Materials, Inc., Taoyuan, Taiwan. The average diameter and length of the MWCNTs with 2–5 layers of sidewalls were 4 nm and 10–12 μm, respectively. The sample of PEG (MW 10,000 g·mol^−1^) was purchased from Sigma–Aldrich, St. Louis, MO, USA.

### 2.2. Preparation of Composite Sensing Film

Four different concentrations of PEG (0.5 g, 1.0 g, 1.5 g, and 2.0 g) were dissolved in DI water to prepare a 100 g PEG solution, and then the mixtures were sonicated for 3 h to achieve uniform PEG solutions. The sensing film was prepared drop-casting to form the bilayer sensor structure of the PEG/MWCNTs composite gas sensor. The top layer was a PEG film, and the bottom layer was MWCNTs film.

### 2.3. Optimum Design of Thermostat for Gas Sensor

**Figure 1:**
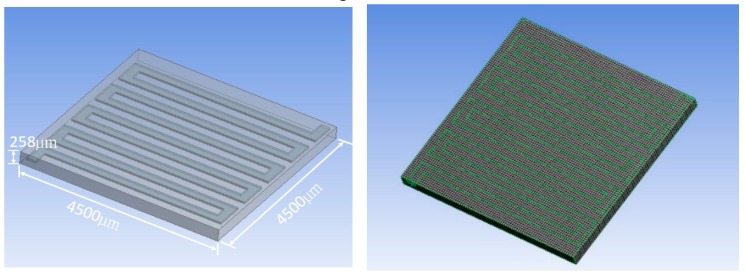
Features of the thermostat and meshed model.

For a sensor equipped with a thermostat for gas sensing, thermal distribution affects the performance of the arranged polymer sensing element. The optimized design for thermal distribution is essential to the thermostat, which ensures each sensor element stays at the same operating temperature. Therefore, the simulation objective is to design and optimize a thermostat for thermal distribution that can minimize the uneven heating problem of a gas sensor. For this reason, different thermostat geometries and heating coil materials were studied to search for the optimized thermal distribution and minimized power consumption [23,24,25,26]. The thermal-electric simulations of the thermostat were developed using a finite element tool ANSYS Multiphysics 17.2. Features of the thermostat and meshed model are shown in Figure 1.

**Figure 2:**
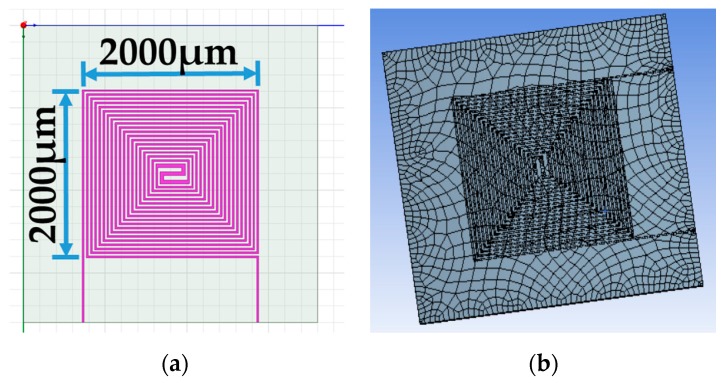
(**a**) Double spiral heater of simulation model. (**b**) Meshed solid model for the thermal-electric simulation.

Furthermore, the optimum geometry of thermostat was implemented to decide the material of heating coil. Four different heating coil materials, including aluminum, platinum, copper, and stainless steel, were selected to evaluate the power consumption of the thermostat at the operating temperatures of 40 °C and 80 °C, respectively. Figure 2 illustrates the simulation model and meshed model.

### 2.4. Gas Sensor Fabrication

**Figure 3:**
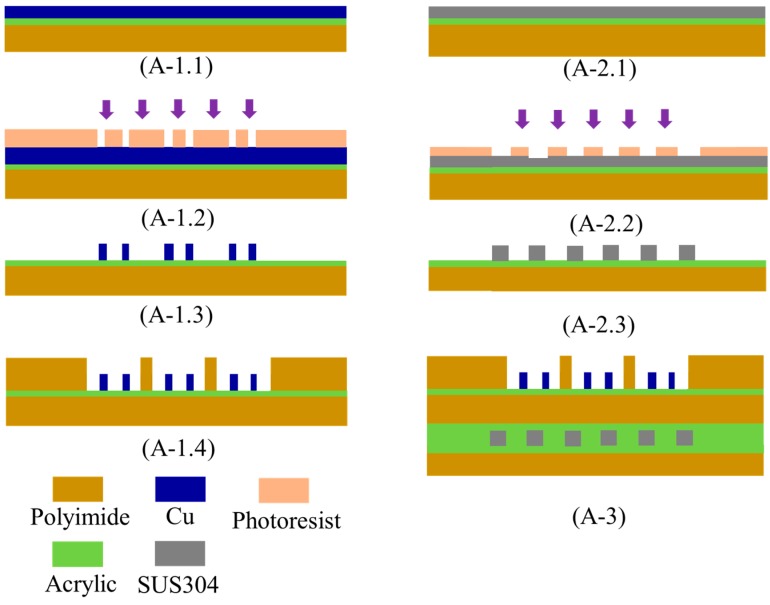
Fabrication process of PEG/MWCNTs gas sensor.

When the optimum geometry and material of the thermostat were determined, the PEG/MWCNTs gas sensor was fabricated by a flexible printed circuit technology. Figure 3 illustrates the proposed fabrication process flow:

(A-1.1) The top layer of the sensor was the interdigitated electrodes (IDEs). Copper with 35 µm thickness was placed on the 50 µm thick polyimide.
(A-1.2)–(A-1.3) The Cu IDEs were fabricated via ultraviolet (UV) lithography and wet etching. Both the width and spacing of Cu IDEs were 220 μm.
(A-1.4) The through-hole machined wells were fabricated via UV lithography and wet etching, then placed around the IDEs, and bonded using acrylic adhesives.
(A-2.1) The selected heating material of stainless steel (SUS304) with 30 μm thickness was placed on the 50 µm thick polyimide.
(A-2.2)–(A-2.3) The geometry of the optimize thermostat was designated as 20 mm × 20 mm. The width and spacing of the thermostat line were 220 and 280 μm, respectively. The double-spiral shape wire was patterned on a substrate using UV lithography and wet etching
(A-3) The fabricated IDEs were aligned and laminated onto the thermostat via acrylic adhesives.

**Figure 4:**
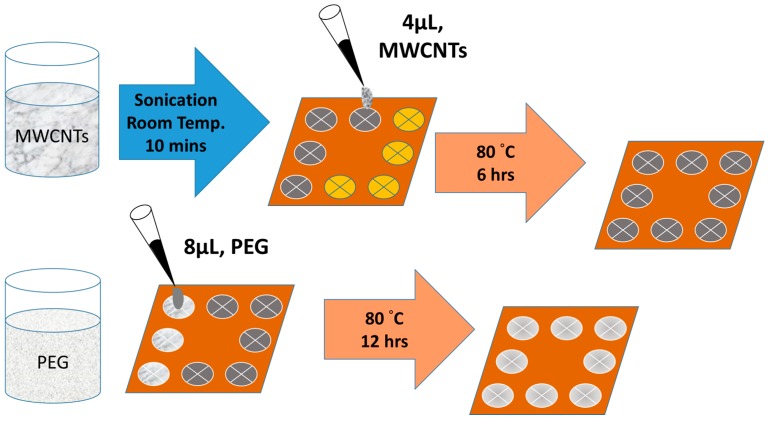
Fabrication process of the PEG/MWCNTs sensing film.

A schematic diagram of the bilayer sensor fabrication process is illustrated in Figure 4. It included two steps regarding the composite sensing film.

(1) In the first step, the MWCNTs was sonicated for 10 min in an ultrasonic bath at room temperature. A 4 μL solution of the MWCNTs dispersion was then deposited on the IDEs by a microjet and then placed in an oven to furnish the MWCNTs film at 80 °C for 6 hours to form the conductive layer.
(2) In the second step, A 8 μL solution of PEG was then deposited on the MWCNTs layer to form the film. The fabricated device was placed in the oven to completely evaporate the solvent at 80 °C for 12 h.

## 3. Experiment

**Figure 5:**
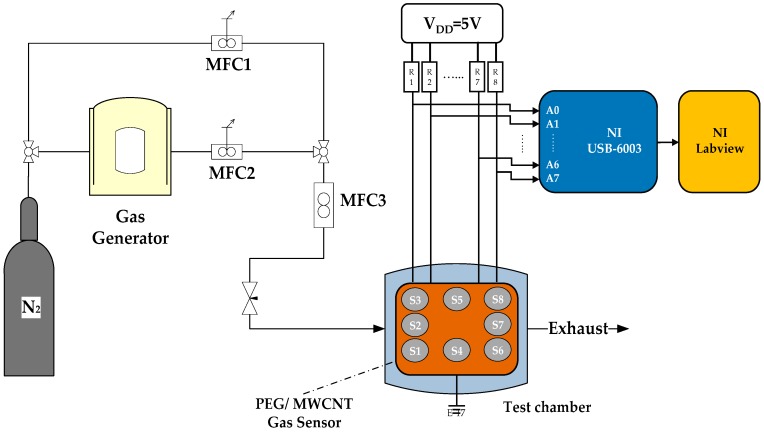
Test instrument of the PEG/MWCNTs gas sensor.

Figure 5 describes the testing apparatus for the gas sensor. The gas generator was a dilution flow system that utilized a standard gas generator (KIN-TEK Analytical, Inc., 670C, La Marque, TX, USA) to evaporate an acetone solvent through a mass flow controller in order to obtain the stable concentration and temperature of acetone vapor. The measurement procedure consisted of several steps in each testing cycle. First, the thermostat was heated. Afterwards, high purity nitrogen (N_2_, ≥99.99%, background gas) was added into the glass test chamber for 30 min to obtain a stable reference baseline. Nitrogen was used as the carrier and gas was purged instead of air to exclude the influence of humidity and oxygen contained in air. Afterward, the evaporated acetone gas (target gas) was passed into the glass test chamber for 500 s, and gas molecules were adsorbed into the sensing film. Then, nitrogen was passed for 500 s for desorption from the sensing film. The flow rates of both the target gas and the background gas were controlled to be under 200 mL/min using a mass flow controller.

The sensor response of the PEG/MWCNTs composite gas sensor to an acetone was investigated under a measurement system. The resistance change of an individual sensor was acquired using a voltage divider method of the fabricated sensor that was installed in a chamber when exposed to acetone. The output voltages of each sensor element were collected via an 8-channel DAQ device (National Instruments Co., USB-6003, Austin, TX, USA). Afterwards, the individual sensor resistance was calculated by Ohm’s law. The collected signal of the resistance eliminated the non-demand trend via post-processing by MATLAB. Finally, the normalized resistance changes (ΔR/R%) was displayed in real time on a personal computer.

## 4. Results and Discussion

### 4.1. Thermostat Performance

The thermal distributions of different type heater coils, including meander, double meander, double spiral, and complex double spiral are listed in Table 1. As a result, the optimum design was the double spiral thermostat which provided the best uniform thermal distribution and minimum thermal variation on the surface of the heating zone.

**Figure 6:**
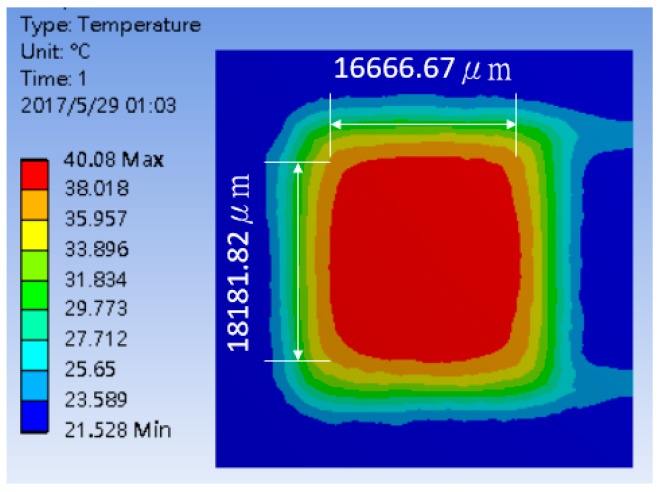
Typical simulation of thermal distribution profile on the surface of the thermostat at 40 °C.

Furthermore, the four different heating coil materials, including aluminum, platinum, copper, and stainless steel, were selected to evaluate the power consumption of the thermostat at the operating temperatures of 40 °C and 80 °C, respectively. The results are listed in Table 2, indicating that the use of stainless steel as the coil material offered the minimum power consumption. The power consumptions at the operating temperatures of 40 °C and 80 °C were about 179.48 and 631.78 mW, respectively. Figure 6 shows the optimum thermal distribution result of the thermostat by using aluminum coil at 40 °C.

**Figure 7:**
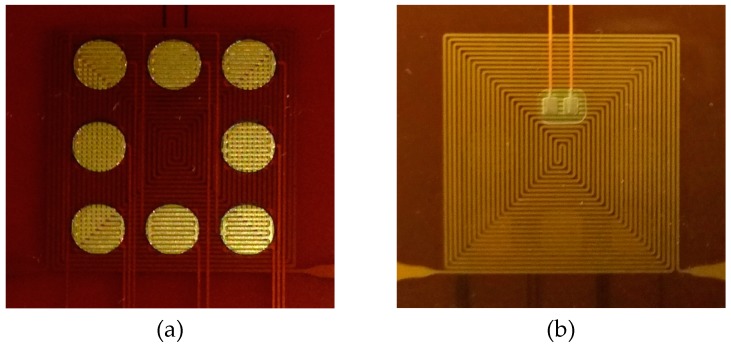
Schematic of the gas sensor equipped with a thermostat. (**a**) front photograph of gas sensor; (**b**) reverse photograph of gas sensor.

**Figure 8:**
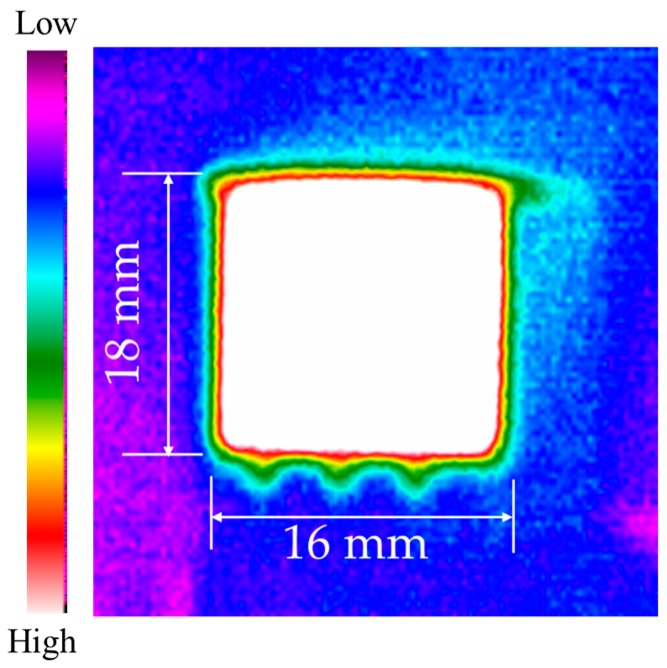
Infrared thermal image of thermal distribution.

The fabricated gas sensor equipped with the thermostat is shown in Figure 7. The thermal distribution of the thermostat was examined using a thermal image camera (Thermoteknix Systems Ltd, MIRICLE 307K-25, Cambridge, UK). The infrared thermal image is shown in Figure 8. Temperature uniformity over the heated area size of the heater corresponds approximately to 18 mm × 16 mm.

### 4.2. Characterizations

**Figure 9:**
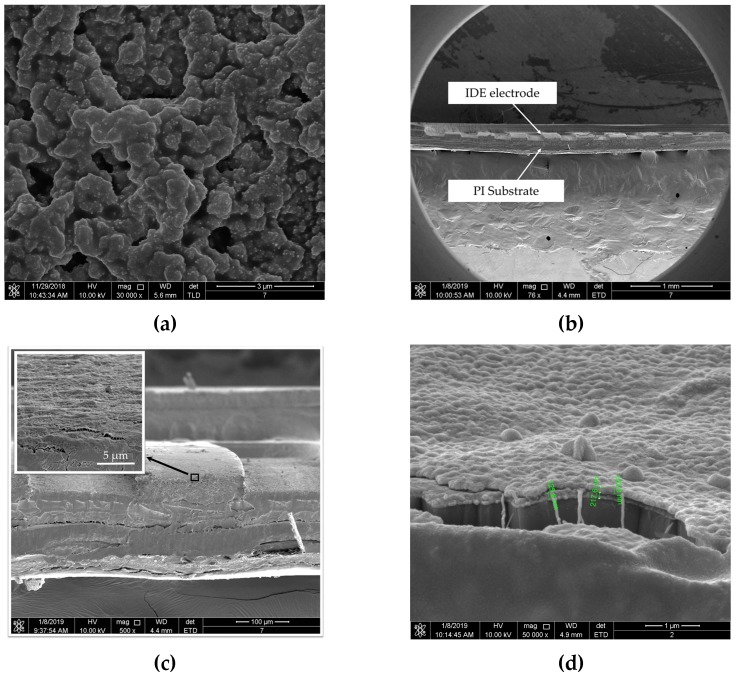
SEM morphologies of (**a**) top-view of PEG/MWCNTs composite film, (**b**) cross-section micrograph of the gas sensor, (**c**) close view of the interface between electrode and PEG/MWCNTs composite film, (**d**) close view of PEG/MWCNTs composite film.

The top-view images of PEG/MWCNTs composite film and cross-sectional morphology of gas sensor were observed by a NOVA NANO SEM 450 (FEI Co., Hillsboro, OR, USA) with 10 kV acceleration voltage. The scanning electron microscope (SEM) morphologies are shown in Figure 9. The thickness of the PEG/MWCNTs composite sensing film fabricated by drop-casting method was about 217.6 nm, and MWCNTs were well wrapped in PEG film.

**Figure 10:**
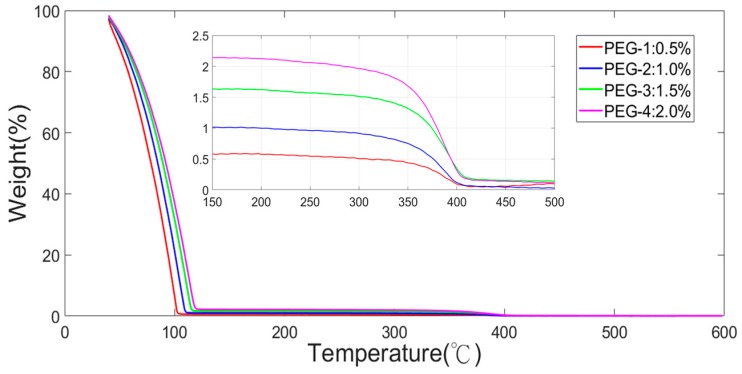
TGA results of four different concentrations of PEG.

The different concentrations of PEG were examined by thermogravimetric analysis (TGA) (Perkin Elmer, Inc., Pyris 1 TGA, Waltham, MA, USA) from room temperature to 500 °C. The TGA results of four different concentrations of PEG are shown in Figure 10, which revealed that the preparation of four different concentrations met the requirement.

### 4.3. Response of Single Film and Composited Film to Acetone Gas

MWCNTs play the role of a p-type semiconductor. MWCNTs that interact with target gas change the conductivity due to the charge transfer between electron-donating or electron-withdrawing molecules via Van der Waals force or donor–acceptor interaction. Moreover, polymer films adsorb gas molecules, and the conductivity is changed by a redox reaction due to the addition or withdrawal of electrons [27,28,29].

**Figure 11:**
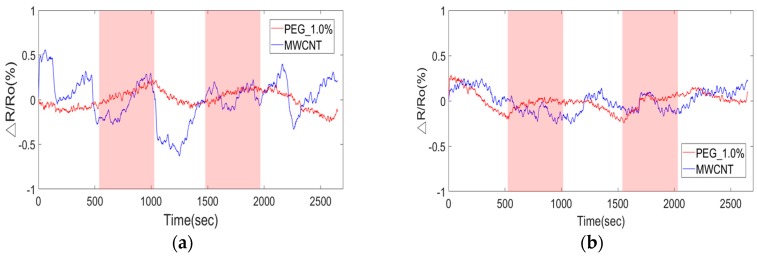
Sensor response of 1.0% PEG and MWCNTs at (**a**) Room temperature and (**b**) 50 °C.

The examined response of a single material sensing film can help to distinguish the intrinsic gas sensing property and interaction. Figure 11 shows that each 1.0% PEG sensor and 60 ppm MWCNTs sensor operated at two different temperatures (room temperature and 50 °C) can detect an acetone gas concentration of 6294.3 ppm. The response of the 1.0% PEG sensor was increased when exposed to acetone and cyclically recovered when exposed to nitrogen. In contrast, the MWCNTs sensor was responsive to both gases. Furthermore, when the operating temperature was 50 °C, the response was more intense than that at room temperature. The results implied that PEG could be the candidate sensing film to detect acetone. Moreover, MWCNTs was responsive to each gas, and the response was increased by increasing the operating temperature.

**Figure 12:**
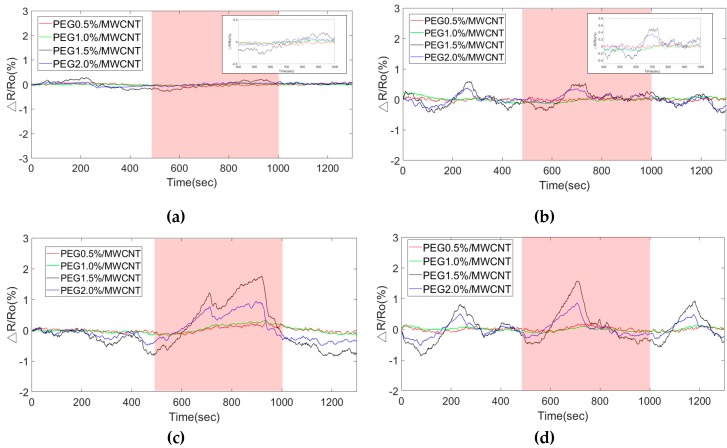
Response of sensor expsoure to 6294.3 ppm acetone at different operating temperatures. (**a**) room temperature (25 °C), (**b**) 40 °C, (**c**) 45 °C, and (**d**) 50 °C.

Figure 12 shows the response of four types of PEG sensing films exposed to acetone vapor of 6294.3 ppm at different operating temperatures, including room temperature (R.T., 25 °C), 40 °C, 45 °C, and 50 °C. When the sensor was operated at a higher operating temperature, the response and signal-to-noise ratio (SNR) were better than those at room temperature. The results exhibited that the PEG chains adsorbed more acetone gas molecules at higher operating temperatures and then enhanced the change of conductivity to dominate the response by a redox reaction due to the addition or withdrawal of electrons [30,31].

### 4.4. Sensor Response of Acetone with Thermal Treatment

**Figure 13:**
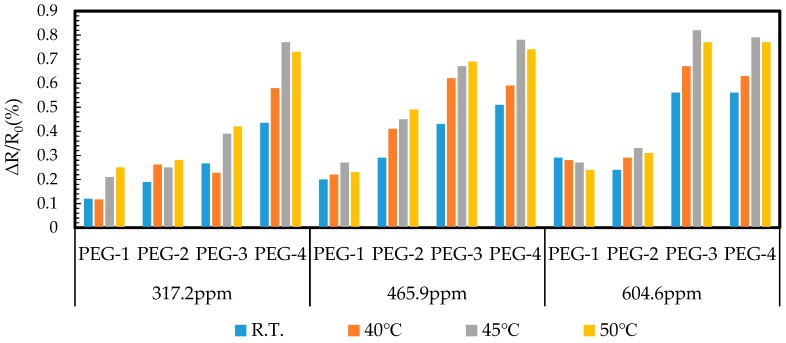
Response of sensor expsoure to different concentrations of acetone at different operating temperatures.

The operating temperature and the acetone vapor concentration deeply affected the response of the PEG/MWCNTs composite sensing material. Therefore, the acquirement of sensor resistance is a straightforward method to observe the phenomenon, which provides much information including the kinetics of atmospheric oxygen adsorption, electron-donating/withdrawing molecules via Van der Waals force, and donor–acceptor interactions [32,33]. To observe the optimum working temperature, the response of the gas sensor fabricated by PEG/MWCNTs was examined as a function of the operating temperature for exposing to 317.2, 465.9, and 604.6 ppm diluted acetone vapors, as shown in Figure 13. The acetone response was increased as a function of the concentration of PEG. This means that the good interaction of PEG surface with the target gas molecules induced the charge transfer in MWCNTs. The response was increased and then achieved its maximum value at 45 °C for each concentration of acetone, possibly because the moderate working temperature provided the thermal degradation of PEG in its side chains to enhance the Van der Waals interaction of PEG/MWCNTs gas sensor surface with vapor molecules [34]. The sensor response of acetone was found to be reduced at a low temperature, which may be due to the weakly dipolar with weak hydrogen bond properties of physically absorb on conducting polymers [35]. Therefore, a high-performance gas sensor is required to identify the proper working temperature for different target gases.

**Figure 14:**
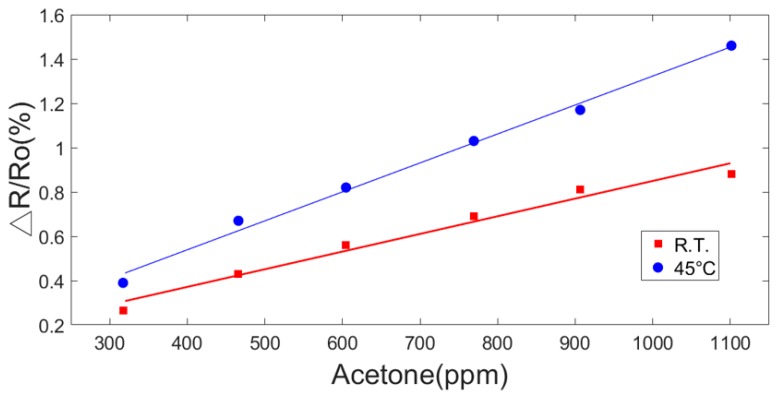
The fitting curves of the sensor response at different operating temperature as a function of acetone concentration (300–1000 ppm).

The response of the 1.5% PEG/MWCNT composite sensing film that operated at two different operating temperatures (room temperature and 45 °C) to exposure to different acetone concentrations (317.2, 465.9, 604.6, 769.3, 906.5, and 1101.3 ppm) is shown in Figure 14. The fitting curves of the sensor response at different operating temperature as a function of acetone concentration. The linear correlation coefficient values of the fitting curves of room temperature and 45 °C, are 0.9758 and 0.9932, respectively.

**Figure 15:**
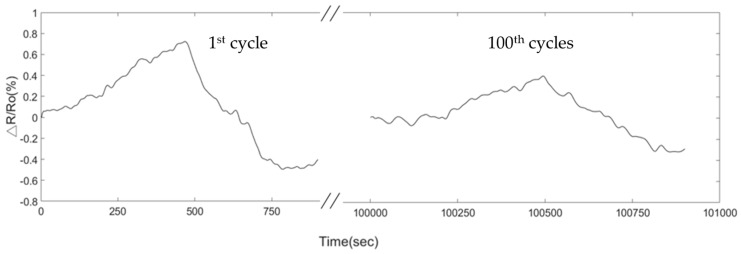
Degradation of the sensor after 100 cycles upon exposure to 604.6 ppm of acetone at 45 °C operating temperature.

The stability test results of 1.5% PEG/MWCNTs composite sensing film at 45 °C in the presence of 604.6 ppm acetone have been examined, as shown in Figure 15. The initial response of the gas sensor is about 0.76% but the response dropped to 0.41% after 100 cycles. The decreased response of PEG/MWCNTs composite sensing film might be the aging issue which due to the incomplete desorption of acetone molecules, water vapor influence and thermal effect. In a practical application, the PEG/MWCNTs composite gas sensor requires calibrating and monitoring the degradation to improve the reliability.

## 5. Conclusions

In this study, an optimum thermostat was created with stainless steel (SUS304) as the double spiral heating element on a polyimide substrate for the application in a gas sensor. Through numerical modeling, the optimum thermostat was designed and fabricated, aimed at improving the uniformity of thermal distribution on the heating zone of the sensor. The PEG/MWCNTs composite film was fabricated on IDEs by drop-casting method.

A comparative study was conducted to investigate response behaviors to acetone of both the single sensing film and composite sensing film. The composite film operating at a modest temperature was found to have some merits over the single film, such as better selectivity and sensitivity. Moreover, four different concentrations of PEG were prepared and formed as the PEG/MWCNTs composite gas sensor where the 1.5% PEG/MWCNTs composite sensing film that operated at 45 °C was found to be more sensitive than other types. Additionally, the sensor response study was carried out for the operating temperature at four different conditions, 45 °C was found to show a better response than other thermal treatment conditions. Furthermore, this kind of gas sensor equipped with a thermostat to immune ambient temperature influence was promising for environmental and industrial applications.

## Figures and Tables

**Table 1:** Selected geometry of the thermostat and related thermal distribution of each thermostat.

Type	Geometry	Thermal Distribution
Meander	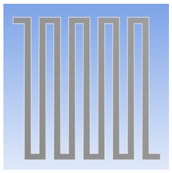	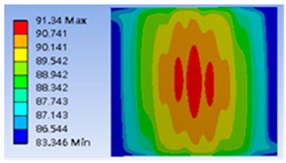
Double Meander	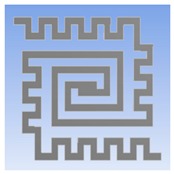	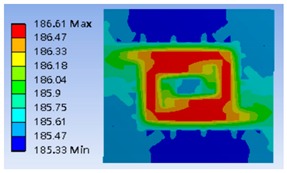
Double Spiral	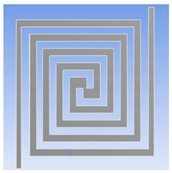	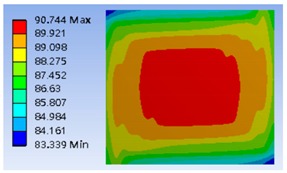
Complex Double Spiral	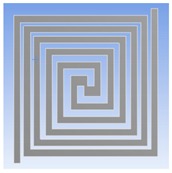	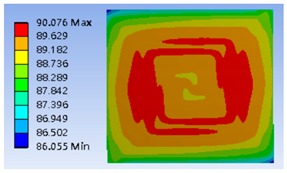

**Table 2:** Power consumption and thermal distribution of each thermostat at 40 °C and 80 °C.

**40 °C**				
Material	**Aluminum**	**Platinum**	**Copper**	**Stainless Steel**
Power (mW)	199.15	190.78	205.76	179.48
Heating Zone (μm^2^)	L: 17,714 W: 16,571	L: 17,777 W: 16,666	L: 17,714 W: 16,581	L: 18,181 W: 16,667
**80 °C**				
Material	**Aluminum**	**Platinum**	**Copper**	**Stainless Steel**
Power (mW)	687.09	666.78	696.62	631.78
Heating Zone (μm^2^)	L: 17,948 W: 17,178	L: 17,948 W: 17,179	L: 17,647 W: 17,058	L: 17,948 W: 17,201
